# Electrochemical ocean iron fertilization and alkalinity enhancement approach toward CO_2_ sequestration

**DOI:** 10.1038/s44183-024-00064-8

**Published:** 2024-05-14

**Authors:** Amir Taqieddin, Stephanie Sarrouf, Muhammad Fahad Ehsan, Ken Buesseler, Akram N. Alshawabkeh

**Affiliations:** 1 Department of Mechanical & Industrial Engineering, Northeastern University, Boston, MA 02115, USA.; 2 Department of Civil & Environmental Engineering, Northeastern University, Boston, MA 02115, USA.; 3 Woods Hole Oceanographic Institution, Marine Chemistry and Geochemistry Department, 266 Woods Hole Rd., Woods Hole, MA 02543, USA.

## Abstract

Achieving net-zero emissions by 2050 requires the development of effective negative emission techniques, including ocean-based approaches for CO_2_ sequestration. However, the implementation and testing of marine CO_2_ removal (mCDR) techniques such as ocean iron fertilization (OIF) or ocean alkalinity enhancement (OAE) face significant challenges. Herein, a novel self-operating electrochemical technology is presented that not only combines OIF and OAE, but also recovers hydrogen gas (H_2_) from seawater, hence offering a promising solution for achieving quantifiable and transparent large-scale mCDR. Experimental results show that the electrochemical OIF (EOIF) can not only increase the concentration of ferrous iron (Fe^+2^) by 0–0.5 mg/L, but also significantly increases the seawater pH by 8% (*i.e*., a 25% decrease in the hydrogen ions concentration). The release of iron (Fe^+2^/Fe^+3^) can be regulated by adjusting the magnitude of the electric current and its form (e.g., pulsed current and polarity reversal), as well as by optimizing the electrode material and geometry. In certain ocean regions, enhanced iron concentrations stimulate the naturally occurring biological carbon pump (BCP), leading to increased phytoplankton growth, CO_2_ uptake, and subsequent export of carbon to the deep ocean. Simultaneously, the system increases seawater alkalinity and the buffer capacity, enhancing CO_2_ solubility and storage in the shallow ocean through the solubility pump. The obtained measurements demonstrate the scalability of EOIF and its ability to operate using solar energy at a lower cost. Overall, the proposed EOIF technology offers a practical, effective, and sustainable solution for addressing climate change on a large scale.

The severe environmental and human health consequences of continuous anthropogenic greenhouse gas emissions require effective and novel solutions. The catastrophic results of climate change, such as the rise in wildfires, floods, and ecosystem disruptions^1–4^ highlight the critical need of urgent and innovative global-scale solutions for carbon dioxide removal (CDR). A target has been set by the United Nations Framework Convention on Climate Change to achieve net-zero emissions by 2050^5–7^, which requires mitigating ~800 Gt carbon dioxide (CO_2_) by 2050^8^. This means that we are required to find possible solutions for avoiding these cumulative emissions of CO_2_ through adapting reduced emission technologies, utilizing effective carbon capture and removal approaches. In fact, achieving net-zero will require not only reducing emissions, but also removing tens of Gt CO_2_ from the atmosphere annually. This scale of removal exceeds the capability of any single existing CDR technology. Relying only in conventional emission reduction techniques, such as transitioning to clean energy technologies (e.g., renewable energy, electric vehicles)^9–16^ will not be sufficient to achieve the 2050 target^5–7^. The development and implementation of net-negative emissions technologies (NET) that are designed to remove CO_2_ directly from the atmosphere and sequester carbon (C) in the ocean for 100 sto1000 years^17–19^,such as marine CDR (mCDR)^20,21^, are crucial toward achieving the goal of net-zero emissions.

CDR is defined as intervention actions that remove CO_2_ from the atmosphere^22^. Designing efficient and cost-effective CDR methods has been one of the primary goals in tackling climate change issues due to their potential to remove a large amount of atmospheric CO_2_. These methods rely on using nature-based technologies for CDR via preserving Earth’s nature and ecosystems^23^. For example, the amount of organic carbon that can be sequestrated in soil from the atmosphere is potentially between 0.79 and 1.54 Gt/year^24^. This approach requires the implementation of sustainable land and soil management protocols to design natural CDR solutions^25^. Enhancing rock weathering could deliver a net CDR of 6 − 30 Mt_CO_2__/year as one natural carbon sequestration solution based on the modeling stuides^26^. This accelerated weathering approach could uptake 45% of the atmospheric CDR for the United Kingdom to achieve its 2050 net-zero emissions target. Alternatively, ocean-based CDR methods are considered promising climate solutions since the ocean can be viewed as a large sink for sequestering the anthropogenic CO_2_ (i.e., up to 30% of CO_2_ emitted by human activities)^20,27^. In terms of capacity, the ocean can store 50 times the amount of CO_2_ present in the atmosphere and 10–20 times more than all plants and soil^24,28^.

mCDR techniques are divided into two main categories: abiotic and biotic methods. Ocean alkalinity enhancement (OAE) is an abiotic approach that is focused on increasing the alkalinity of the seawater to increase the buffer capacity of the ocean and enhance air-sea gas exchange via the solubility pump^29–33^. Generally, increasing the pH of seawater increases the saturation state for carbonate minerals^18,34^, which are used by many marine organisms to form shells and skeletons depending on several factors (e.g., temperature, salinity)^29–31^. As increased levels of CO_2_ enter the ocean and react with water, forming carbonic acid and decreasing ocean pH, OAE would cause additional atmospheric CO_2_ removal to counter the deleterious impact of ocean acidification. A modeling study conducted in the Bering Sea demonstrated that the mCDR efficiency could increase to 96% after three years of total alkalinity enhancement^33^. On the other hand, ocean iron fertilization (OIF) is a biotic approach for mCDR in which iron (Fe) is supplied^35,36^ as an essential micronutrient in certain areas of the ocean where it limits primary production (see Fig. 1a). Therefore, even small amounts of Fe can greatly stimulate the net growth of phytoplankton^7,35–45^. As the phytoplankton population expands and photosynthesis increases, dissolved CO_2_ in the ocean decreases, and the uptake of atmospheric CO_2_ increases^46,47^. Through a biological carbon pump (BCP), the organic C derived from sinking heterotrophs creates organic-rich “marine snow” particles that sink, exporting 3% of the organic C down to the deep ocean and sea floor, where it would be sequestered for hundreds to thousands of years^48,49^. Several field experiments and studies have shown the promising potential of increasing mCDR using OIF, suggesting that the ocean could sequestrate gigatons of additional atmospheric CO_2_^7,38,41,44,50–53^ if OIF were applied to large areas of the ocean that have high macronutrients (such as nitrate and phosphate) and low Fe. For example, enhanced carbon export into the deep water has been achieved by field experiments of natural iron fertilization at the Southern Ocean^54^. The significant potential of OIF as a large-scale negative emission technology and natural CDR solution has recently brought substantial attention from the scientific community and policymakers to advancing OIF techniques^7,17,21,36,50^.

While both OIF and OAE represent promising mCDR solutions, testing and the implementation of these two approaches have been challenging. For example, regulating and monitoring the Fe release in the ocean through repeated additions over a long term is arduous^7,43,55^. The main advantage of OIF as an mCDR approach is that the Fe concentrations needed to stimulate phytoplankton growth are low compared to other nutrients. In natural systems, the Fe:C ratio in marine phytoplankton is 1:100,000 or even less (*i.e*., less Fe)^56^, making this one of the most cost-effective mCDR approaches. Traditional OIF methods operate by releasing bulk quantities of Fe in the ocean via ships/airplanes as solid particles or in aqueous form (see Fig. 1a)^36,43^. Prior experiments have shown that most of the added Fe through FeSO_4_ is lost due to rapid oxidation, precipitation, and scavenging by sinking particles, and/or is considered not readily bioavailable^55^. Additionally, the requirement of having a ship on site, or possible delivery by aircraft for the Fe release limits the supply to shorter-term additions. However, for mCDR deployments, longer-term and repeated Fe additions over larger areas may be more effective. Increasing the bioavailability of the supplied iron over time and through repeated deployment to the phytoplankton requires a clear understanding of the iron speciation (e.g., ferrous versus ferric)^57^. While ferrous (Fe^+2^) is more soluble in seawater than ferric (Fe^+3^), both forms can be taken up by marine phytoplankton^39^. The bioavailability and the uptake of these species by phytoplankton depend on the reductive thermodynamic processes and seawater conditions (see Sutak et al. −2020 for more details^39^). It is important to note that Fe^3+^ is predominant in oxygenated and neutral pH surface waters since Fe^+2^ is oxidized under these conditions. Larger concentrations of Fe^+2^ can be formed as the pH decreases (*i.e*., more acidic) compared to Fe^+3 58^. Understanding the interdependent relationship between the iron speciation, oxygen content, and pH level is crucial for enhancing iron bioavailability.

Several electrochemical techniques have been developed and proposed as mCDR solutions^59–64^. These techniques are mainly designed to increase the alkalinity of seawater to capture CO_2_^59–64^. For instance, an earlier study demonstrated the potential of using electrochemical systems that convert CO_2_ into calcium carbonate by inducing local changes in the pH at the cathode^63^. Recently, an electrochemical system comprising of bismuth and silver electrodes was tested to capture and release chloride ions from seawater, leading to changes in the overall pH through CO_2_ removal^59^. These proposed electrochemical systems use inert anodes, which produce acids or chlorine gas (Cl_2_) during electrolysis leading to unfavorable conditions (e.g., a decrease in the pH of seawater) at the anode. Further, these systems use inert metal as anode and cathode, such as bismuth and silver, which makes scaling expensive. Finally, the previously developed electrochemical systems^59–64^ tend to rely only on OAE as a mitigation strategy for mCDR without realizing the promising potential of OIF.

Motivated by utilizing ocean-based CDR solutions to tackle climate change at a global scale, a novel EOIF approach is proposed that combines OIF and OAE to improve the ocean capacity for carbon storage and CDR sequestration. Our approach to advancing these systems as mCDR solution is focused on employing Fe/Fe-producing anodes to eliminate the production of acids at the anode in traditional electrochemical OAE systems and replace it with Fe release^65–68^. In this case, the electrochemical system implements both OIF and OAE techniques at the anode and cathode, respectively, where the cathode can be made from naturally abundant cost-effective materials, such as carbon. The proposed EOIF approach overcomes major limitations of the existing electrochemical techniques for mCDR. The design and the concept of the proposed EOIF technology are discussed. Experimental evidence on its ability to regulate the Fe release in seawater and increase the pH is discussed. Additionally, the EOIF system is designed to extract H_2_ from the seawater as detailed in the following section. Overall, the EOIF represents a promising sustainable, self-operating, and effective solution for mCDR.

## Results

### EOIF technology

**Fig. 1|:**
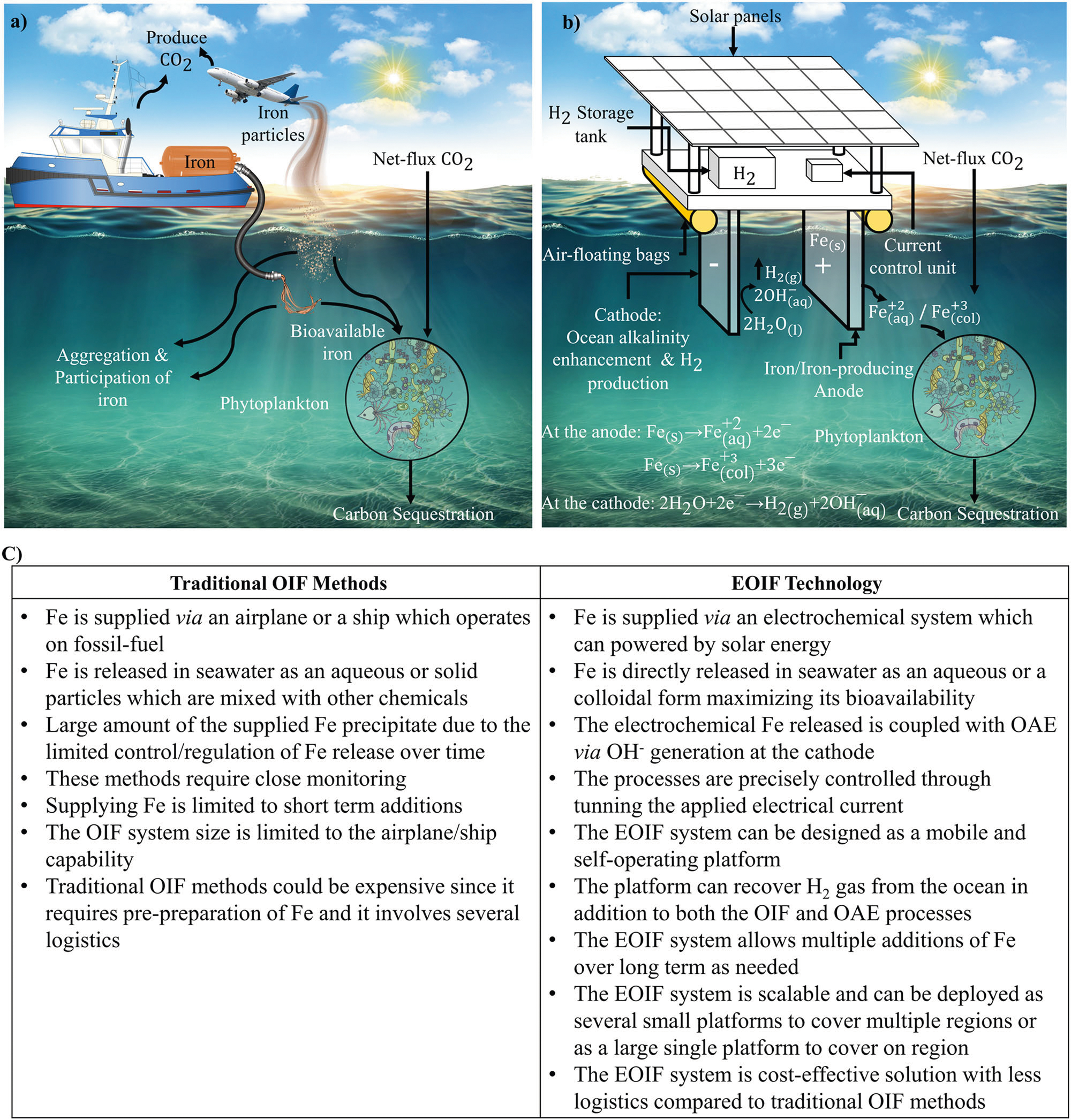
Schematic of OIF approaches. **a** traditional OIF using boat or airplane to release Fe in seawater in pre-prepared aqueous or solid particles forms, **b** the proposed EOIF technology, which is envisioned as a mobile offshore platform that is powered by solar energy panels, and **c** summary of the benefits of EOIF compared to traditional methods.

The proposed EOIF relies on releasing Fe in the ocean *via* electrochemical reactions at the electrode as shown in Fig. 1b. When an electrical current is applied across the electrodes, ferrous ions, Fe^+2^, will be directly released in aqueous phase from a solid Fe/Fe-producing anode^65,69,70^. The ferric ions, Fe^+3^, can be released in a colloidal form. The electrochemical reactions of Fe^+2^ and Fe^+3^ at the anode have standard potential reactions equal to 0.44 V and 0.037 V, respectively, and given as^58^:

(1a) $${Fe}_{\left(s\right)}\to {Fe}_{\left(aq\right)}^{+2}+2e^{-},$$

(1b) $${Fe}_{\left(s\right)}\to {Fe}_{\left(col\right)}^{+3}+3e^{-}.$$

The main advantage of the EOIF technology is its ability to regulate and control the release of Fe according to Faraday’s law where the amount of released Fe(m) is given as:

(2) $$m=\frac{I\;\times \;t\;\times \;M_{w}}{n\;\times \;F}$$

where I is the electrical current, t is the duration of the reaction, $M_{w}$ is the molecular mass of the Fe, n is the number of participating electrons, and F is Faraday’s constant. Clearly, m can be directly controlled by tuning the values of I and t. In this manner, the flux of Fe in the ocean can be controlled over time and space to maximize the bioavailability and distribution of Fe in the ocean and avoid any oversupply leading to Fe saturation in the ecosystem which could lead to precipitation of siderite or eutrophication style effects.

Further, the reactions at the cathode can also be designed to boost and enhance the alkalinity of the ocean. At the cathode, seawater splitting takes place as follows:

(3) $$2H_{2}O+2e^{-}\to H_{2(\;g)}+2{OH}_{\left(aq\right)}^{-}.$$

The produced $H_{2}$ can be collected and recovered for later usage as fuel such as powering the EOIF system. More importantly, the released ${OH}^{-}$ in the ocean will increase the ocean’s alkalinity without the need to add additional minerals to seawater. The enhanced alkalinity and pH will help increase the ocean’s capacity to intake atmospheric CO_2_ and de-acidify the ecosystem. The cathode material selection is not limited to a specific choice if it ensures optimized chemical reaction kinetics. For example, the cathode material can be made of carbon as a cost-effective choice^71,72^. Here, several cathode materials were tested. The Fe and OH^−^ generated in-situ from the electrodes transfer directly into the seawater in their dissolved aqueous form, eliminating the need for additional chemicals (e.g., SO_4_^2−^ in case of FeSO_4_ additions). Specifically, the proposed OAE approach does not generate unnecessary acid components during alkalinity enhancement, unlike traditional OAE approaches^33^.

It should be noted that secondary competing reactions can take place at electrode surfaces which can lower the efficiency of the EOIF system. For example, depending on the system pH, hydrolysis or oxidation of ${Fe}_{\left(aq\right)}^{+2}$ can lead to the formation of either aqueous ferric $\left({Fe}_{(aq}^{+3}\right)$ or ferrous hydroxide ${(Fe(OH)}_{2(s)})$ precipitates^58,73^. Chloride ions can also react at the anode to form chlorine gas or are adsorbed at the anode by the surface polarization^74,75^. Additionally, oxygen evolution reaction (OER) can also occur at the anode as follows:

(4) $$2H_{2}O_{\left(l\right)}\to O_{2(g)}+4H_{\left(aq\right)}^{+}+4e^{-}.$$

The generated $O_{2}$ gas can be collected and stored or can end up leaving the ocean’s surface in the atmosphere. In general, Fe oxidation is typically the dominant reaction at the anode. Furthermore, the electrical current can be adjusted to prevent other competing reactions. At the cathode, secondary reactions can also occur such as converting the formed ${OH}^{-}$ to $O_{2}$ gas. Therefore, it is important to control the environment of the reactions to reduce unnecessary secondary reactions at the electrodes.

**Fig. 2 |:**
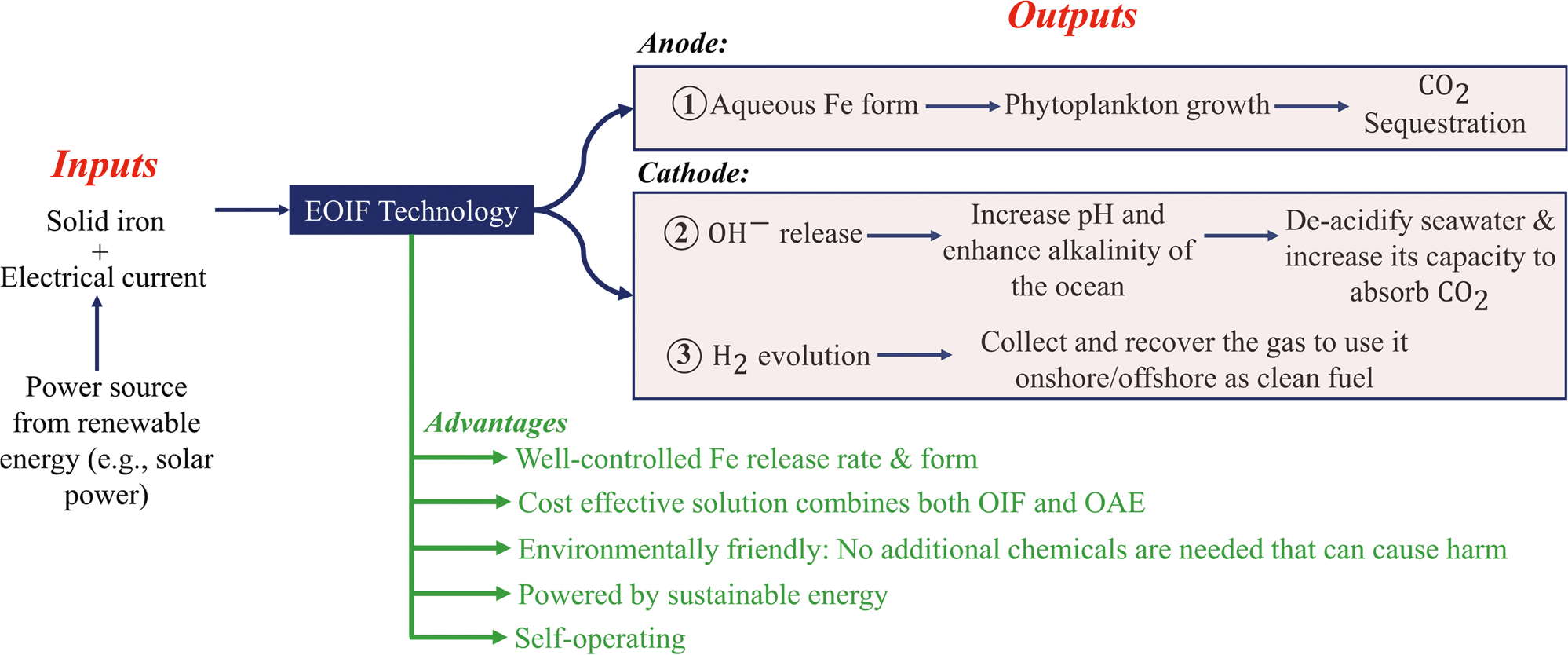
Summary of the EOIF technology. Schematic of the inputs, outputs and advantages.

Overall, the EOIF system can be deployed as a mobile offshore platform, as shown in Fig. 1b. The key features of the EOIF platform are summarized in Fig. 2. Produced gases, such as H_2_, are considered as secondary products (refer to Fig. 2). A key innovation of the EOIF is its ability to control the rate of Fe release according to Faraday’s law where the release flux is directly proportional to the current at the electrodes. The system can be self-operating in offshore environments, which lowers the total cost and carbon footprint compared to traditional OIF methods that require a shipor an airplane for operations (Fig. 1a). Further, it represents a cost-effective solution given it has a simple design that combines both OIF and OAE concepts for tackling climate change.

### Electrochemical iron release in seawater

**Fig. 3 |:**
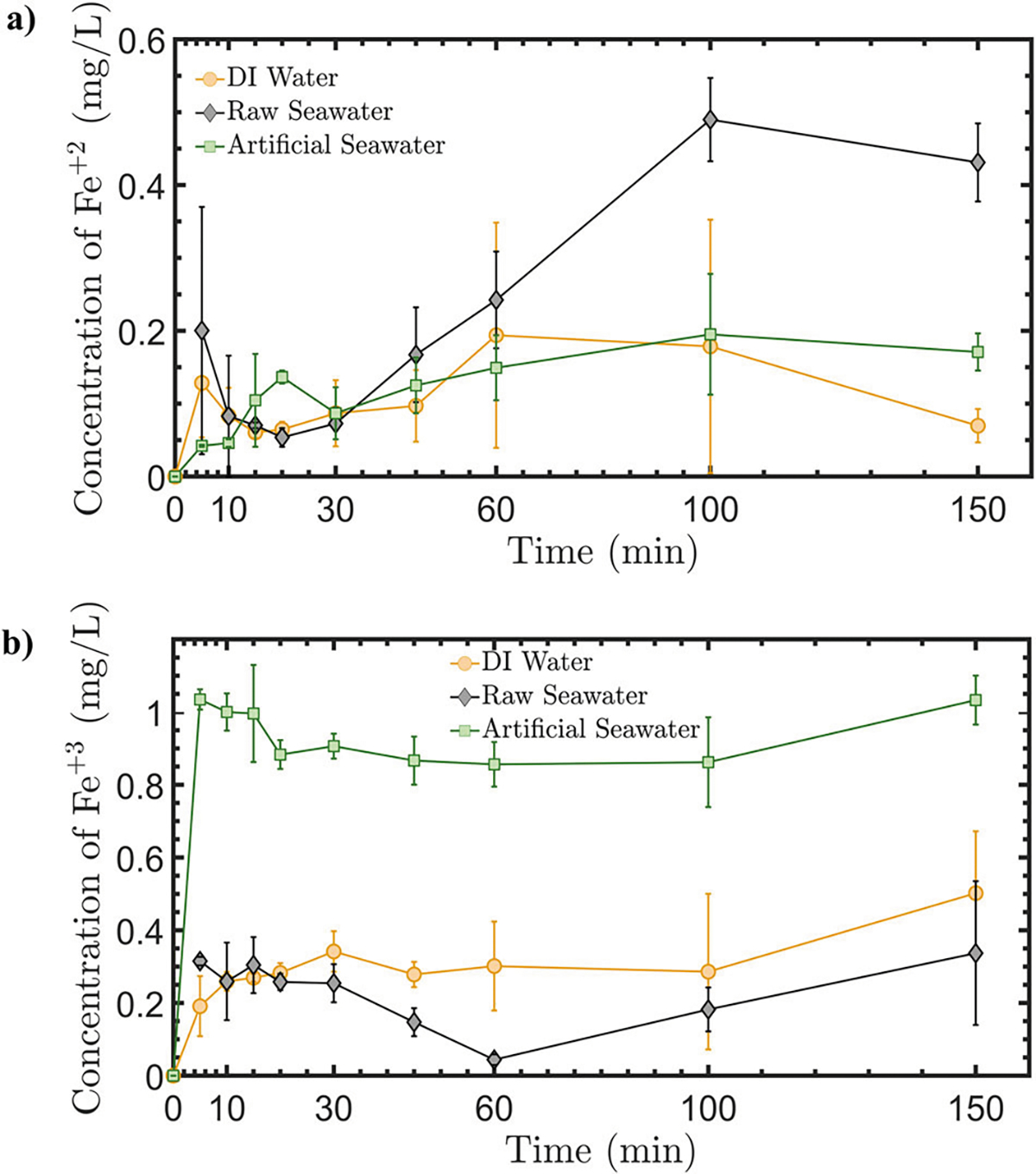
Measured concentrations of the electrochemically released iron as a function of time in various water samples and under 160 mA electrical current. **a** Fe^+2^ and **b** Fe^+3^ concentrations.

Various experiments were performed to demonstrate and verify the release of Fe in seawater using EOIF. First, the release of Fe^+2^ and Fe^+3^ was investigated in artificial seawater, deionized (DI) water, and natural seawater. The natural seawater was collected from the shore labs at Woods Hole Oceanographic Institute, as indicated in the method sections. These specific experiments were performed in 1 L beakers where Fe plates were used as both anode and cathode. The current in this set of experiments was set to 160 mA. Figure 3 shows the concentration of Fe^+2^ and Fe^+3^ in artificial, DI, and natural raw (i.e., unfiltered) seawater as a function of time. Although the concentration profiles are nonlinear due to the exchange between different chemical forms of Fe (*i.e*., Fe^+2^ and Fe^+3^)^65,69^, an increase in the Fe concentrations was observed as a function of time due to electrochemical processes. Specifically, the initial concentrations of both Fe^+2^ and Fe^+3^ in the artificial seawater are zero since it was prepared using DI water without any iron traces. Immediately after 5 min of applying the electrical current and initiating the electrochemical reactions (*i.e*., first collected data point in all experiments), the presence of different forms of Fe (Fe^+2^/Fe^+3^) was observed in both artificial and DI sweater samples.

The Fe concentration ranges are with in the typically measured range of released Fe in natural waters^76–78^. It should be noted that the iron concentration in the ocean is on the scale ~5.6 × 10^−5^ mg/L (or 1 nmol/L). The obtained measurements indicate a maximum value of ~0.5 mg/L of Fe^+2^ in raw seawater (Fig. 3a), and ~1 mg/LofFe^+3^ in artificial seawater, as shown in Fig. 3b. This value can be lowered by decreasing the current intensity. Here, a relatively large current was applied to ensure the released Fe is within the detection limit of the measuring instrument. More important, the tests were performed in 1 L systems. In the actual ocean, the volume of water will be significantly larger, and the concentration of Fe will thus be more diluted for the same released amount of Fe where it can be expected to reach the target range of ~5.6×10^−5^ mg/L. Figure 3a initially shows a decrease in Fe^+2^ concentration in raw seawater due to an increased Fe^+3^ concentration (as shown in Fig. 3b). However, the Fe^+2^ concentration subsequently increases after the concentration of Fe^+3^ is stabilized.

The amount of Fe^+2^ concentration in the reactor increases almost linearly as a function of time during 30–100 min of the reaction. However, the Fe^+3^ concentration decreases between 30–60 min and increases again between 60 and 150 min. The decrease in the concentration of Fe^+3^ indicates the transformation of Fe^+3^ to Fe^+2^ between 30–60 min. After 60 min, the concentration of Fe^+2^ becomes large enough, and, therefore, part of the Fe^+2^ is transformed into Fe^+3^. While the presence of Fe^+3^ is essential to assist in absorbing Fe^+2^ by phytoplankton^39,79^, it is important to keep it under control since it displays low solubility in the water at near neutral pH. Our results show that the electrochemical approach can offer the potential to optimize the Fe^+3^ concentration as a function of time in seawater (*i.e*., specifically a minimum concentration of Fe^+3^ at time 60 min in raw seawater experiment). Figure 3 shows that the ratio of Fe^+3^ to Fe^+2^ concentration is about 0.65 at 150 min of the electrochemical reaction in the raw seawater sample (*i.e*., Fe^+3^ and Fe^+2^ concentrations are about 0.3 and 0.45 mg/L, respectively). This means that Fe^+2^ concentration is about 60% of the total released Fe in raw seawater sample at 150 min. At 60 min, the ratio of concentration is about 0.15 indicating an optimized reaction time at which the released Fe^2+^ is maximized compared to Fe^+3^ (*i.e*., Fe^+3^ and Fe^+2^ concentrations are about 0.036 and 0.24 mg/L, respectively) where the concentration of Fe^+2^ is about 87% of the total released Fe in the raw seawater sample. This suggests that the electrochemical reactions for releasing Fe can be controlled and stopped at a specific time once optimized conditions are achieved. In other words, the amount and the chemical nature of the released Fe can be controlled over time to maximize the bioavailability of the generated Fe by the electrodes.

Lower Fe^+2^ and higher Fe^+3^ concentrations were observed in artificial seawater and DI water. For DI water, a low total amount of Fe is released due to its low ionic conductivity as an electrolyte compared to artificial and raw seawater which is rich with various ions. The low ionic conductivity reduces the efficiency of the Faradaic reactions leading to lower Fe flux from the electrodes. Demonstrating the electrochemical Fe release in DI water establishes a control experiment base to further investigate the effect of adding minerals for the synthesis of artificial seawater. Two different chemical compositions of artificial seawater were tested (see Table S1). The electrochemical release of Fe in both composition samples shows the same concentration of Fe^+2^ and Fe^+3^ as a function of time (see Figure S3). The presence of biological species and complex chemical compounds in raw seawater assist in keeping the electrochemically released Fe in solution, leadingtohigherFe^+2^ andsignificantlylowerFe^+3^ concentrations compared to the artificial seawater. This is confirmed by running the electrochemical experiments under the same conditions using filtered and UV oxidized seawater (*i.e*., UV and 100 μm filtered samples). The results (see Figure S4) show that lower Fe^+2^ concentrations are detected in filtered seawater samples toward the end of the experiments. Thus, a larger concentration of insoluble Fe^+3^ is observed in artificial seawater due to the absence of natural organic matter compared to raw natural seawater, which is important to the solubility of released Fe. Specifically, Fig. 3 illustrates that the concentrations of Fe^+3^ in artificial seawater are approximately three times higher than that in the raw natural seawater sample, while the Fe^+2^ concentration is approximately two times lower. In the following sections, various approaches were investigated to control electrochemical Fe release in artificial seawater, including manipulating the ratio of Fe^+3^ to Fe^+2^ concentrations. It should be highlighted that the expected concentration of Fe^+3^ and Fe^+2^ in natural seawater is significantly less and larger, respectively, compared to the artificial seawater results based on the results in Fig. 3 and the previous discussion. In the following sections, the experiments focus on investigating Fe production in artificial seawater, allowing more experimental control given their chemistry rather than natural raw seawater.

### Regulating electrochemical iron release

**Fig. 4 |:**
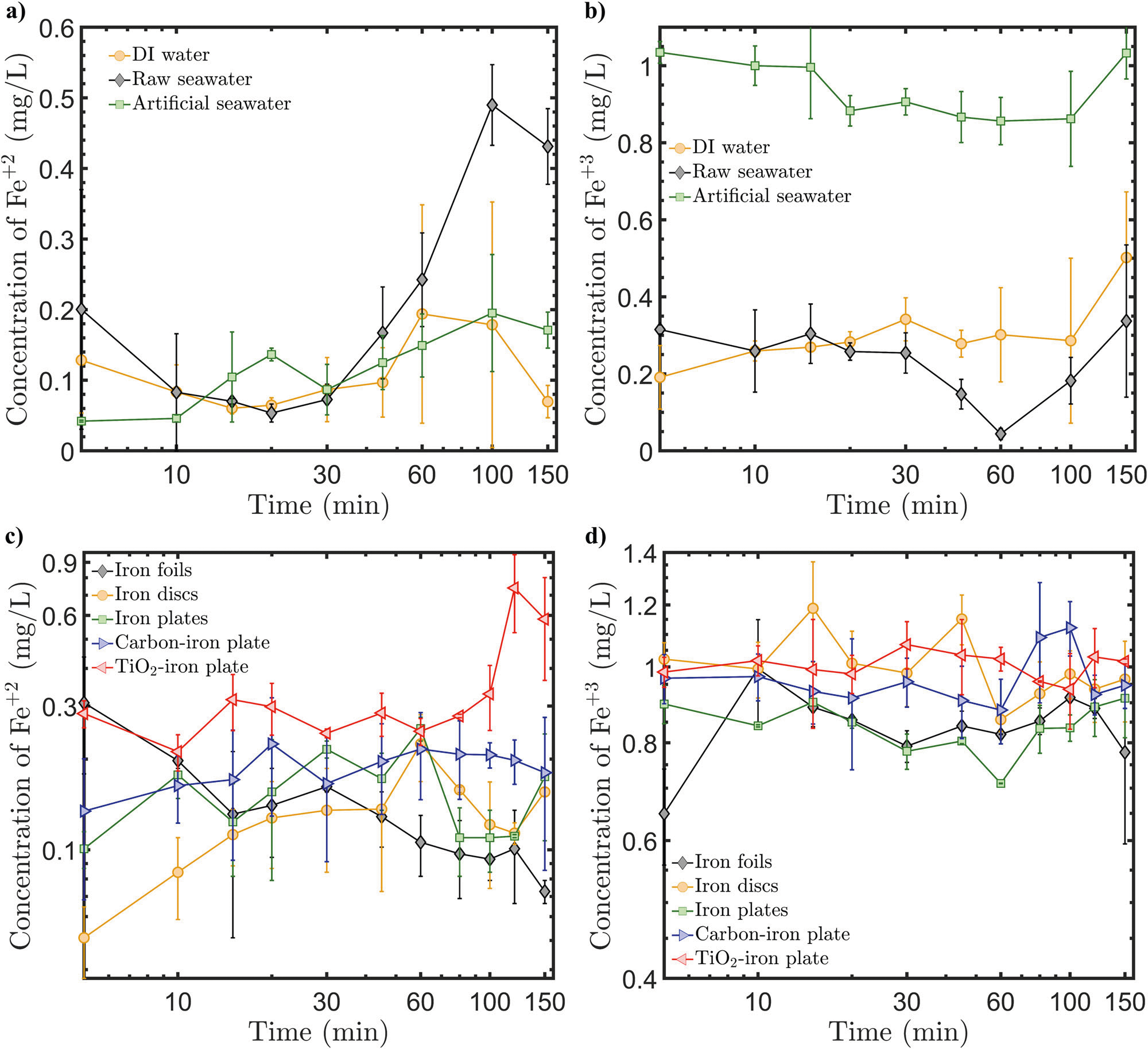
Measured concentrations of the electrochemically released Fe forms as a function of time under 160 mA electrical current. **a** and **b** Fe^+2^ and Fe^+3^ concentrations, respectively, for various electrodes, **c** Fe^+2^ (solid lines) and Fe^+3^ (dashed lines) concentrations under current pulsing conditions, and **d** Fe^+2^ (solid lines) and Fe^+3^ (dashed lines) concentrations under polarity reversal conditions.

According to Eq. 2, the primary parameter, which controls the release of Fe in the EOIF system is the current. As the magnitude of the current increases, the released Fe increases in the system. However, a large increase in the electrical current could result in Fe precipitation which leads to reduced Fe^2+^ in solution. Therefore, changing the electrode geometry and material type was investigated as a first protocol to optimize the Fe release before controlling the electric current. Figures 4a, b show the measured concentration of Fe^+2^ and Fe^+3^ using various electrode geometries and materials (see Methods section for specific details). The results show that using Fe discs for both the anode and cathode leads to lower Fe^2+^ concentration over time compared to using Fe plates or foils. This is because the Fe discs have smaller surfaces compared to the other geometries, so the surface available for the reactions is smaller, producing less Fe. The Fe foil electrodes result in a decreasing profile of Fe^2+^ concentration over time. Since the foil is very thin, it conducts the electrical current very well. Therefore, the initial time it takes for the foil to release a large amount of Fe^2+^ is due to its high efficiency in conducting the current. However, the produced Fe^2+^ starts transforming into Fe(OH)_2_ and precipitates because OH^−^ production at the foil cathode is fast and efficient. Given that the reactor size is small and the electrodes are adjacent to each other, parasitic reactions such as the formation of Fe(OH)_2_ can take place at the electrodes. However, the focus in this paper is on the primary reactions, and it should be emphasized that the final EOIF system would be engineered to reduce any secondary reactions, such as increasing the distance between the electrodes to prevent the reaction of the generated OH^−^ with the Fe^+2^ at the anode or vice versa.

In addition to studying the electrode geometry, different cathode materials were investigated to assess their cost-effectiveness and impact on the efficiency of Fe release. In the previous results, the Fe release was investigated in symmetrical electrochemical systems where the cathode and anode were made from Fe electrodes. Here, while keeping the same Fe plate anode, three different cathode materials were tested, including Fe plate, carbon bar, and titanium mixed metal oxides (Ti/MMO) mesh. The measurements show that using Ti/MMO as a cathode led to higher concentrations of Fe^2+^ compared to the Fe plate and carbon cathodes. This indicates that Ti/MMO has higher electro chemical efficiency due to its mesh structure which allows for regulating the Fe release by forming a complex electric field domain with the anodic plate (*i.e*., both anode and cathode have different geometry, which results in a less effective electric field compared to using electrodes with same dimensions and geometry). The concentrations of Fe^+3^ over time have the same values for the various electrodes (see Fig. 4). The carbon cathode shows an intermediate efficiency in terms of generating Fe^+2^ over time. The concentration profile of Fe^+2^ for the carbon-based cathode system lies between the Titanium-based and the Fe-plate cathode systems. Given the low cost of carbon-based materials, using carbon material as a cathode would be an optimum design in terms of cost and Fe release efficiency.

To investigate the efficiency of the Fe release as a function of the electric current, the current was varied from 40 to 220 mA using either a carbon or Ti/MMO cathode. Figure S5 shows that increasing the current from 40 to 100 mA increased the overall concentration of Fe^+2^ for the carbon-based cathode system. The further increase to 220 mA led to a minimal increase in Fe generation compared to lower current values. Similar trends were observed while using a Ti/MMO cathode, as shown in Figure S6. This is due to the large and fast Fe generation at the anode. The rapid generation of Fe at larger currents makes it harder for the released Fe to have enough time to dissolve in the seawater and therefore Fe^+2^ will have a minimum concentration. Alternatively, pulsed current and polarity reversal techniques^80,81^ can be respectively used for manipulating the current and switching between the electrodes (*i.e*., anode to cathode or vice versa) to regulate and maximize the Fe release and its bioavailability. Pulsed current is applied by switching the current on/off for different time cycles. Figure 4c shows that Fe^+2^ production can be completely suppressed by pulsing the current every 10 min (*i.e*., 10 min ON followed by 10 min OFF) for either carbon- or titanium-based cathode systems. If the current is pulsed every 5 min, a slight generation of Fe^2+^ was observed as a function of time. The Fe^+3^ concentrations are larger for pulsed current every 5 min interval compared to 10 min interval. Overall, current pulsing over 5 min intervals using a Titanium-based cathode shows that the Fe^+2^ concentration increases as the reaction time evolves while the Fe^+3^ concentration decreases. This is attributed to providing enough time through pulsing for the Fe to dissolve into the seawater allowing more soluble Fe^+2^ formation over insoluble Fe^+3^. Pulsing the current or other time-dependent current applications can be used to regulate the Fe release over time. The efficiency of the current pulsing technique needs be better demonstrated in larger systems beyond 1–2 L reactors.

The polarity reversal approach is based on periodically switching the cathode to the anode and the anode to the cathode to regulate the electrochemical kinetics. Figure 4d shows the Fe concentrations using the polarity reversal approach. Fe^+2^ concentrations are higher when 10 min intervals of polarity reversal are applied. This is because a 10 min interval allows the Fe to be produced for 10 min and then the production is stopped for another 10 min giving more time for the Fe to dissolve in the seawater compared to the 5 min interval procedure. Furthermore, there is a point in time at which at which the Fe^+2^ concentration reaches the maximum (~0.6 mg/L), and the Fe^+3^ concentration is at a minimum (~0.9 mg/L). This point is at a 55 min mark of the 10 min polarity reversal curve using a Ti/MMO cathode. Optimization of the EOIF control parameters would be needed to take full advantage of the Fe bioavailability and regulate its release in the seawater.

## Discussion

### Solar-powered EOIF and techno-economic assessment

**Fig. 5 |:**
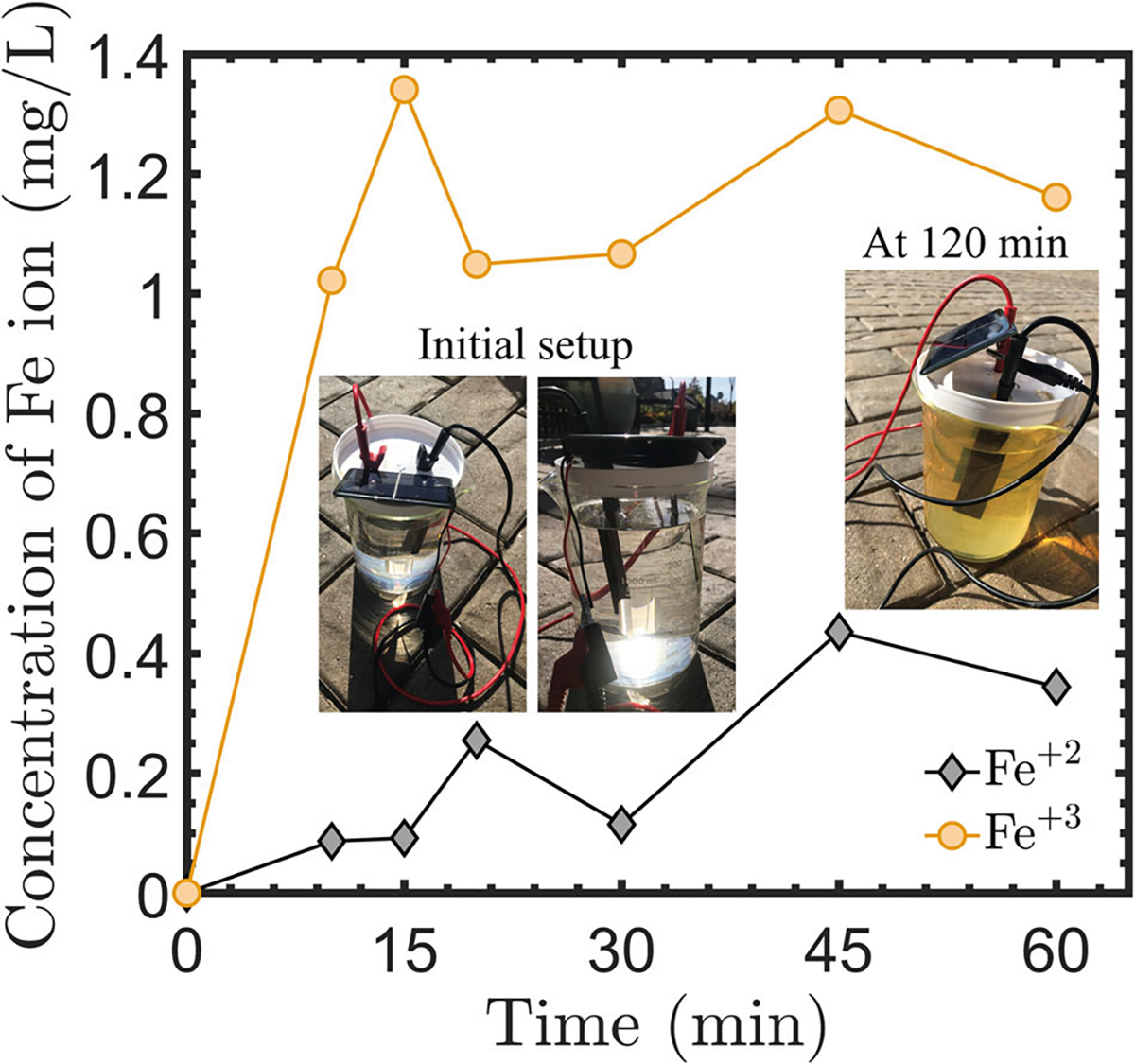
Solar-powered EOIF. Measured Fe^+2^ and Fe^+3^ concentrations when the current is supplied using a solar panel.

As shown in Fig. 2, the EOIF technology promotes sustainability where the input of electrical current can come from a renewable energy source such as solar power. To investigate the practicality of using solar energy to power the electrochemical reactions of the Fe release, small-scale experiments were performed as shown in Fig. 5. The solar-generated electrical current using a small panel (see Methods section for details) produced both Fe^+2^ and Fe^+3^ as expected. The results in Fig. 5 demonstrate that the concentration of the Fe (Fe^+2^ and Fe^+3^) increases over time. A large presence of Fe^+3^ was observed, that tuned the artificial seawater into a yellow color since this Fe form is insoluble. We also observed the generation and release of H_2_ gas bubbles at the carbon cathode as shown in the recorded video (see the Supplementary Materials). The solar-harvested electrical current can be tuned and controlled using current control units or by adjusting the incident solar energy.

To demonstrate the cost feasibility of powering the EOIF platform using solar power, the financial cost of this technology was estimated and compared it to the other reported CDR approaches as shown in Table 1. First, the required amount of Fe to increase the ocean Fe concentration was set as the target of the techno-economic assessment based the experimentally reportedvalues^46^. It is assumed that the ferrous Fe concentration (Fe^2+^) should be increased by 1 nM in seawater (*i.e*., an estimated value within a range to prevent supplying an unnecessarily large amount of Fe beyond the saturation growth limit of phytoplankton^46^). Assuming this 1 nM of Fe^2+^ should be supplied over a surface area of 100 × 100 km (roughly one degree of latitude/longitude) and depth of 30 m, then the volume of the sweater will be 3 × 10^11^ m^3^ or 3 × 10^14^ L. The needed mass of ferrous Fe, *m*, is given as the product of the targeted supplied Fe concentration times the volume of the seawater such that *m* = 3 × 10^5^ mole or 16.75 tons: Using Faraday’s law (*i.e*., Eq. 2), the amount of needed solar energy can be estimated in terms of the electrical current to supply 3 × 10^5^ moles of Fe^+2^. Assuming that the Fe amount is supplied over 5 years (*i.e*., *t* = 15:77 × 10^7^ s), then the total needed current for the reactions, given that *n* = 2 and *F* = 96; 485 C/mole, will be 367 A. This current can be obtained using a PV cells system of series panels of 20 kW where each panel can provide a current of ~6–8 A. Therefore, it is estimated that the platform requires about 70 connected solar panels of a total equivalent area of 1400 sq ft, which could cost about $55,000 based on commercial online prices of solar panels. It should be noted that the needed 16.75 tons can be supplied by running several platforms at different locations in the ocean to complete fertilization in less than five years. These calculations show the feasibility of the EOIF process. The amount of the generated OH^−^ during the five years process, and the usage of 367 A, can be determined using Eq. 1 as well. Given the time and current from the designed Fe release, the amount of released OH^−^ will be around 10 tons or 2 nM over the targeted seawater volume. This amount will help in increasing and enhancing the alkalinity of the ocean. Thus, the invested $55,000 in solar panels can provide 16.75 tons of Fe for OIF and 10 tons of OH^−^ for OAE. The potential form CDR is greater for OIF than OAE since the response and C growth and export efficiencies can be 1:1000 and higher *via* OIF compared to 1:1 efficiency *via* OAE^17^. Based on these efficiencies, the amountofremovedCO_2_fromtheatmospherecanbeestimatedtobe16,760 tons based on the combined addition of 16.75 tons of Fe and 10 tons of OH^−^ using the EOIF processes.

The estimated average price of iron is about ~ $90/ton. The total cost of the needed iron is about ~ $1,500. It can be assumed additional ~ $3,000 (double of the iron electrode’s cost) will be needed to construct the EOIF platform and occupy it with the various equipment such as a power supply to control the current and carbon-based cathode. Thus, the total cost of the EOIF platform can be rounded up to $60,000, which includes the cost of the solar panels, iron electrodes, platform frames, cathodes, and other equipment. This cost represents the price of materials and energy of the EOIF processes. For simplicity, the logistic cost is not considered in this analysis assuming a self-operating platform (*i.e*., in this case the cost of the logistic operations will mainly depend on the cost of the deployment of the EOIF platform in the ocean). Using the previously estimated amount of sequestered atmospheric CO_2_ (16,760 tons) and the total cost of the EOIF platform ($60,000), the estimated price of mCDR *via* EOIF is about $3.58/tons of CO_2_. This techno-economic assessment shows that EOIF is a cost-effective approach compared to other mCDR methods where their financial cost is estimated to range between $10-$1000/tons of CO_2_^18,82,83^. Even if the financial cost of EOIF is assumed to be doubled due to the logistics or any hidden effectiveness parameters (*i.e*., the cost becomes ~$7.16/tons of CO_2_ as an extreme case), the cost of the EOIF approach is still less that the other mCDR methods (<$10/tons of CO_2_).

This feasibility analysis shows the effectiveness of the EOIF method. Previous experiments of OIF released about 1–3 ton of Fe over couple of weeks and batch size up to 300 km^2^,^44^. The previous theoretical estimation suggests that the EOIF approach releases almost 8 times the Fe and regulates it over longer periods of time (e.g., 5 years) instead of a couple of weeks. This allows for monitoring the biogeochemical response of the ecosystem over time, and also meets the marine demand of 10–100 ton of Fe^7^. In terms of cost, earlier experimental studies showed that the power requirement for electrochemical mCDR methods such as OAE is 1.8–2.3 MWh/tCO_2_^33,84–86^. This is a large energy consumption, which could require building costly offshore wind turbines^33^. The high energy demands of traditional OAE techniques prevents effectively implementing and testing these techniques offshore. Overall, the EOIF system is a promising novel, sustainable, and cost-effective technology that provides a mCDR solution combining both OIF and OAE approaches.

### Demonstrating scalability of EOIF

**Fig.6 |:**
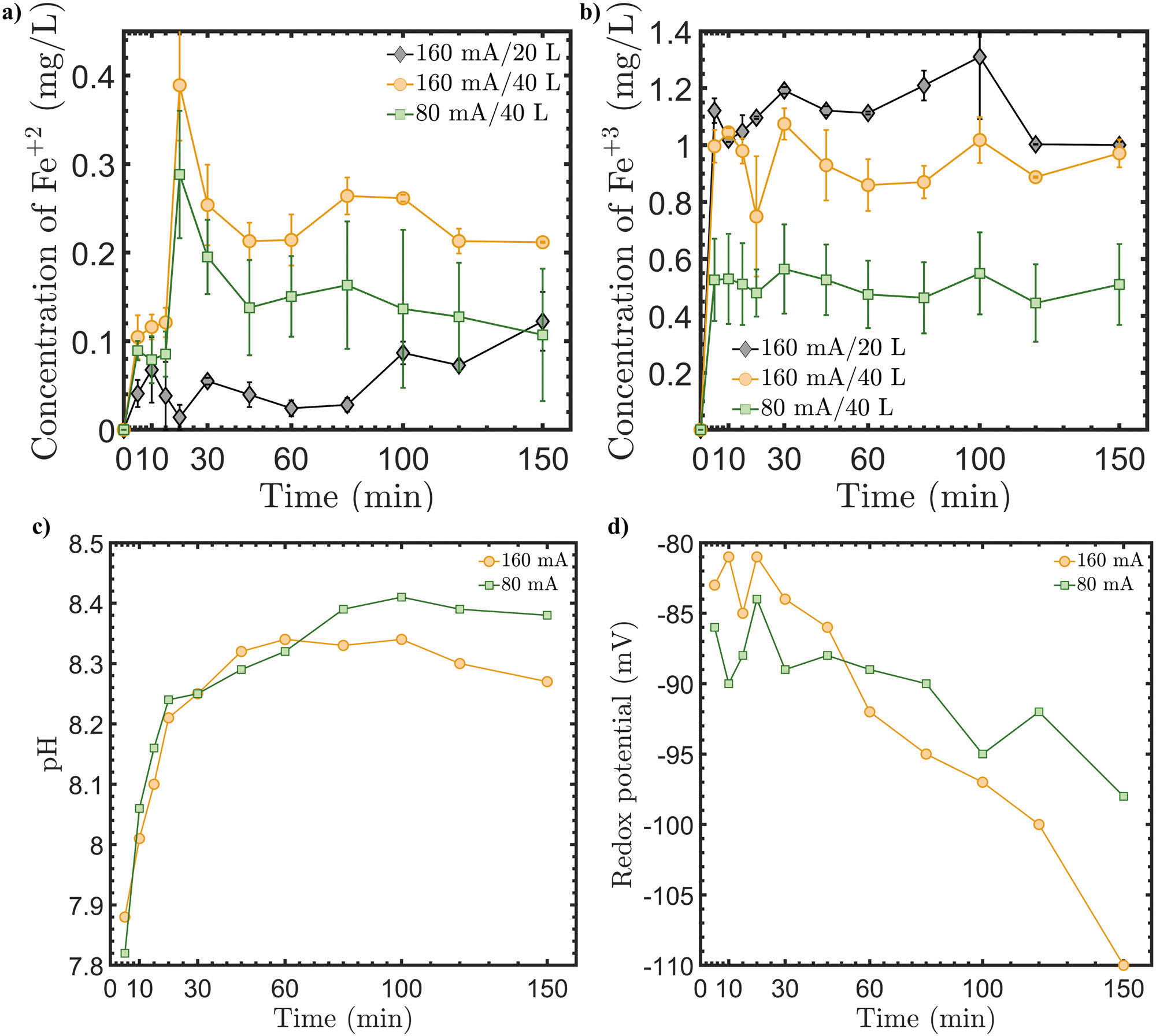
Experimentally measured results during the electrochemical Fe release in large tanks. **a** and b concentrations of Fe^+2^ and Fe^+3^, respectively, as a function of time for various seawater tank sizes and currents, c pH evolution in 40 L seawater tank, and d the oxygen redox potential in the 40 L tank using 80 and 160 mA currents.

The experimental results presented here are based on small-scale testing. It will be important to demonstrate the scalability of the proposed EOIF technology by testing larger systems. In larger systems, it is expected that the Fe concentrations become less (*i.e*., the same amount of released Fe mass at a given current but in larger seawater volume) and easier to control. Specifically, the Fe^+3^ concentration should be lower since the volume is increased, and natural ligands enhance Fe^+2^ solubility. To demonstrate that, the Fe release and pH variations were tested in larger tanks, as detailed in the Method section. Figure 6 shows the results for testing the EOIF system in 20 and 40 L tanks. First, the measured concentrations show that the Fe^+2^ is found to be less in the 20 L tank versus the 40 L tank under 160 mA, whereas the trend is the opposite for Fe^+3^ concentration. This supports our earlier observation about the competition and exchange between Fe^+2^ and Fe^+3^ forms in seawater. When the tank size is larger, the volume of seawater is larger and therefore the released Fe^+2^ will have sufficient vicinities to dissolve in the water without transferring into Fe^+3^. Thus, the effective amount of the released Fe^+2^ can be increased and scaled with larger systems.

Next, an increase in Fe *via* EOIF can be achieved by tuning the applied current. Figure 6a shows that the Fe^+2^ concentration over time is proportional to the applied current for the same tank size (*i.e*., 40 L). As the current increases from 80 mA to 160 mA, an increase in the Fe^+2^ concentration was observed while maintaining the same concentration profile as a function of time for both currents. Furthermore, the Fe^+3^ concentration decreases by almost 50% upon reducing the current from 160 mA to 80 mA. This is caused by the smaller electrochemical Fe flux using reduced potential (80 mA), which allows insoluble ferric Fe precipitation. The concentrations of Fe^+3^ and Fe^+2^ using the 20 and 40 L tanks represent promising scalability features of the EOIF technology. Finally, Fig. 6c, d show the time-dependent changes in the pH and redox potential of the seawater in the 40 L tank under 80 and 160 mA currents, which are relevant to alkalinity enhancement. The results show the pH of the seawater significantly increasing over time as expected for both currents by ~0.6. The redox potential also became more negative as time evolved indicating the formation of OH^−^ in the seawater. It should be highlighted that the pH under 80 mA is slightly higher than the pH under 160 mA, which can be justified by the possible consumption of OH^−^ to form Fe(OH)_2_ in a secondary reaction as the current increases as previously discussed. Overall, the larger volume experiments validated the scalability of the EOIF technology toward achieving well-designed OIF and OAE processes.

### Final remarks

EOIF technology represents a promising solution to increase the ocean capacity for CO_2_ removal and enhance primary production in the ocean based by combining both OIF and OAE concepts, in addition to potentially allowing for hydrogen recovery from the ocean (H_2_ quantification was not tested here). The electrochemical release of Fe and OH^−^, which are controlled by Faraday’s law, demonstrate an engineered route to regulate the Fe release rate, its bioavailability, and the pH of the seawater. Unlike the traditional methods of OIF, the EOIF releases the Fe without the need to add any additional chemical compounds and offers the ability to regulate the release over time by controlling the applied current. Experimental results show that the electrochemical Fe release in natural raw seawater produces a concentration range of 0.05 mg/L < Fe^+2^ < 0.5 mg/L, which can be lowered by either increasing the tank size or lowering the electric current. The detected Fe^+2^ in artificial seawater is almost 50% less than the detected concentration in natural seawater due to the transformation of Fe^+2^ into Fe^+3^ in artificial seawater (*i.e*., Fe^+3^ concentration is almost 5 times higher in artificial seawater than natural seawater). This is attributed to the absence of natural organic matter and minerals in artificial seawater that help in stabilizing the electrochemically released Fe in natural seawater resulting in higher soluble Fe^+2^ concentration over the insoluble Fe^+3^. Furthermore, the release of Fe in seawater can be controlled and optimized by varying the electrode materials and geometry. It was demonstrated that using a carbon-based cathode with a Fe plate anode can be a cost-effective option with an intermediate efficiency in terms of the released Fe form (*i.e*., Fe^+2^ versus Fe^+3^). Using a Ti/MMO mesh cathode with an Fe plate anode result in maximum efficiency by producing the largest Fe^+2^ concentrations. The EOIF system offers several ways to control the Fe release and its form, such as current pulsing and polarity reversal. Our experiments revealed that Fe^+2^ production can be suppressed under certain electric current pulsing or polarity reversal conditions.

The applied current can be obtained from solar power. Solar-powered EOIF technology is a feasible and cost-effective solution to increasing the Fe concentration by either deploying several small EOIF platforms or a large platform to achieve the desired concentrations. In addition, the EOIF system demonstrates the potential to be scaled up, by adjusting the applied current and the volume and geometries. The key parameter that controls the Fe release is the magnitude of the applied electrical current. Therefore, lower Fe^+3^ concentrations can be optimized, leading to potentially higher bioavailable Fe^+2^. In addition to the measured Fe concentration, the pH increased over 150 min by almost 0.6 for the 20 and 40 L tanks. The decreasing redox potential as a function of time indicates the release of OH^−^ in the system. While the EOIF technology represents a potential solution for OIF and OAE studies and deployments, additional investigations and field testing are required to achieve an optimized design, durability, and control systems for open ocean use. Comparing the bench scale experiments with the field studies is crucial for a reliable comparison between the EOIF technology and other OIF approaches at the larger scales. For example, the possible secondary reactions at the electrodes, such as the production of chloride gas and Fe(OH)_2_ formations should be studied. Specifically, integrated analysis of the scale-up parameters of the EOIF should be investigated in future work. This requires investigating the effect of the ocean mixing in both horizontal and vertical directions on the iron transport and the surface flux of CO_2_. In addition, the mixing analysis can help in understanding the potential increase in the carbon export, and the potential risk of precipitation of Mg and Ca salts caused by localized high carbonate ion concentrations. Future studies can also help in quantifying the extent of OAE *via* EOIF in the field and the associated potential of the CO_2_ sequestration. Overall, the results are intended to bring EOIF to the attention of the scientific community as a potentially effective approach to reduce atmospheric CO_2_ and thus tackle climate change at larger scales.

## Methods

### Analytical and testing methods

During all tests, the Fe^+2^ and Fe^+3^ concentrations were quantified (Note: only the dissolved forms of iron were measured). All tests were performed at room conditions (*i.e*., 25 °C and 1 atm). Batch experiments were carried out in 1 and 2 L beakers. Large tests (*i.e*., 20 and 40 L) were in 40 L plastic tanks. Analysis of Fe^+2^ and Fe^+3^ was determined by UV-VIS spectrophotometer (SHIMADZU UV-1800) at 510 nm wavelength, using the 1–10 phenanthroline analytical method^87^. To quantify the total dissolved Fe, a sample of 0.5 mL taken from the beaker was filtered by a 0.2-micron filter, then 0.25 mL of 10% hydroxylamine hydrochloride solution was added to reduce the dissolved Fe to Fe^+2^. 1 mL of acetic acid buffer solution (20 g of ammonium acetate added to 25 mL of acetic acid in 100 mL of water) was added to adjust the pH from 3 to 5, and an addition of 1 mL of the 1–10 phenanthroline monohydrate (1 g/L) was added consecutively to the sample. The same procedure is followed to determine the concentration of Fe^+2^ using UV-VIS spectrophotometer at wavelength 510 nm except that no hydroxylamine hydrochloride was added. Finally, the concentration of Fe^+3^ was determined by taking the difference between the total dissolved Fe and Fe^+2^ concentrations.

The calibrations of the Fe^+2^ and Fe^+3^ concentrations performed by the spectrophotometer are shown in Figure S1 and Figure S2. Continuous measurements of pH and oxygen reduction potential (ORP) at top of the reactor were carried out using portable electrochemical pH probe meters with an estimated error of 0.1 in the pH values. At the beginning of each experiment, the testing beakers/tanks were washed and brushed several times with DI water and sulfuric acid (10% by weight) to remove any contaminants of the surface. All collected samples were filtered using 0.2-micron membranes (13 mm syringe filters with Millex-LG hydrophilic PTFE membrane before analyzing the Fe concentrations. All experiments of the figures in the main manuscript were repeated three times to obtain proper averaging and estimate the potential errors. The electrical current was applied across the electrodes using an Agilent E3612A DC power supply by setting it to constant current output. The solar-powered experiment was carried out using a 60 mm x 90 mm solar panel of 1 V and 400−500 mA outputs.

### Materials for testing

Solid Fe electrodes in different shapes were tested ranging from a plate, disc, and foil to investigate the effect of electrode geometry on the Fe release in seawater. The plate electrode is 19.05 mm wide × 101.6 mm long and weighs about 12 grams. The disc Fe electrode is 24.26 mm in diameter with 99.9% purity. The foil electrode was cut from a 0:1 × 100 × 1000 mm Fe sheet with 99.995% purity. A piece was cut that has a width and length equal to the plate electrode dimensions. For the carbon electrode, carbon bars were used with dimensions of 100 × 20 × 5 mm made by Eisco labs. Ti/MMO electrode consists of IrO_2_ and Ta_2_O_5_ coating on Titanium, with a diameter of 2.9 cm and 2 mm in thickness with a purity of 99%.

Artificial seawater electrolyte was prepared in the lab. The chemical compositions of the prepared artificial seawater samples are detailed in Table S1. These chemical compositions are determined based on previous work^88^. The chemical minerals and compositions were added to DI water to synthesize the artificial seawater samples. The reported experiments in this manuscript are based on Sample 2 composition shown in Table S1 except for Figure S3 where both artificial seawaters from Sample 1 and 2 were tested and compared. The natural unfiltered and filtered seawater samples were collected at Cape Code shore in Massachusetts, United States of America.

## Supplementary Material

SI

SI Video PPT

## Figures and Tables

**Table 1 |:** Comparison of the financial costs of different mCDR technologies

Technology	Financial cost (US$/tons of CO_2_)
Ocean liming (OAE technology)	72–159^18^
Electrochemical weathering (OAE technology)	14–190^18^
Accelerated weathering of limestone (OAE technology)	10–40^18^
Direct air capture (OAE technology)	100–1000^18^
Traditional OIF *via* airplane delivery	7–415^84^
Traditional OIF *via* ship delivery	9–1502^84^
Self-operating solar-powered EOIF (combined OIF and OAE)	3.58–7.16

## Data Availability

Additional data supporting the analyses and results of this study are available in the Supplementary Information. Additional generated data are also available from the corresponding author upon request.
