# Supplementary material for: Electrochemical ocean iron fertilization and alkalinity enhancement approach toward CO2 sequestration
Source: NPJ Ocean Sustain. Author manuscript; Available in PMC 2024 Dec 13. (PMC11643492; doi:10.1038/s44183-024-00064-8)
Supplement: SI [file NIHMS1995114-supplement-SI.pdf]

Supplementary Information: Electrochemical Ocean Iron Fertilization and Alkalinity Enhancement Approach Toward CO2 Sequestration

Amir Taqieddin[1], Stephanie Sarrouf [2], Muhammad Fahad Ehsan[2], Ken Buesseler[3], Akram N. Alshawabkeh[2,*]

[1] Department of Mechanical & Industrial Engineering, Northeastern University, Boston, MA 02115

[2] Department of Civil & Environmental Engineering, Northeastern University, Boston, MA 02115

[3] Woods Hole Oceanographic Institution, Marine Chemistry and Geochemistry Department, 266 Woods Hole Rd., Woods Hole, MA, 02543, USA

This supplementary information files contains 6 supplementary figures and 1 table.

[*] Corresponding Author, e-mail: a.alshawabkeh@northeastern.edu

Table S1 Chemical composition of the prepared artificial seawater electrolyte

**Sample 1**		
**Chemical**	**Mass (g)**	**Density (g/L)**
NaCl	234.6848	58.6712
$MgCl_2$	49.5585	12.38963
Na_2SO_4	39.252	9.813
$CaCl_2$	1.1854	0.29635
KCL	6.6685	1.667125
$NaHCO_3$	1.995	0.49875
NaBr	0.9597	0.239925
$SrCl_2$	0.2597	0.064925
NaF	2.91	0.7275
**Sample 2**		
**Chemical**	**Mass (g)**	**Density (g/L)**
NaCl	103.188	25.64588
$MgCl_2$	9.83424	2.46374
$MgSO_4$	13.2168	3.306475
$CaCl_2$	4.56107	1.145442
KCl	2.927	0.737813
$NaHCO_3$	0.85	0.218644
NaBr	0.329	0.089713

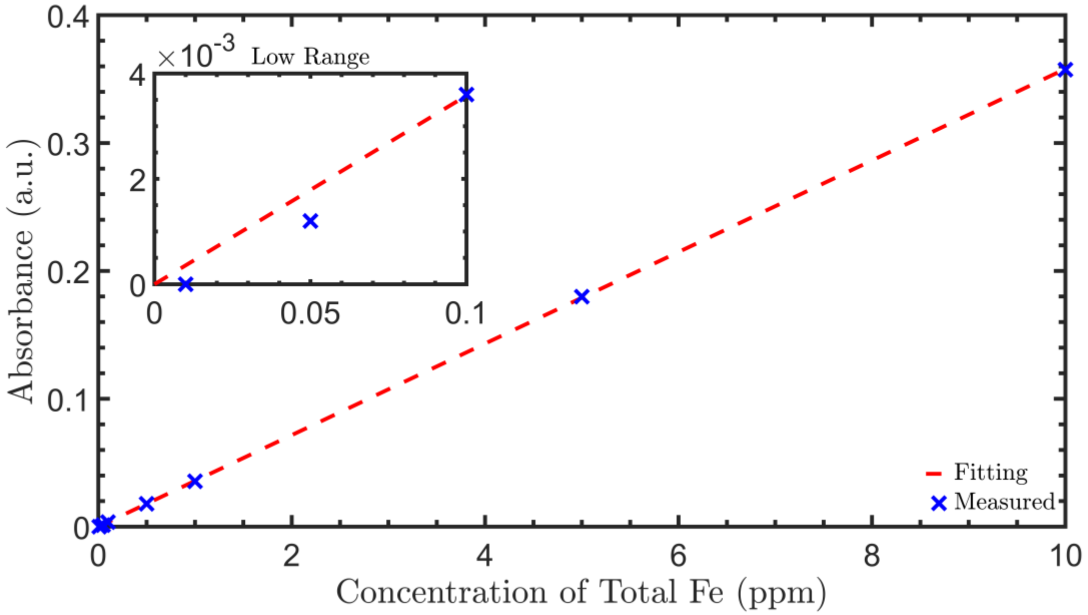

Figure S1. Calibration of total Fe for analysis using spectrophotometer.

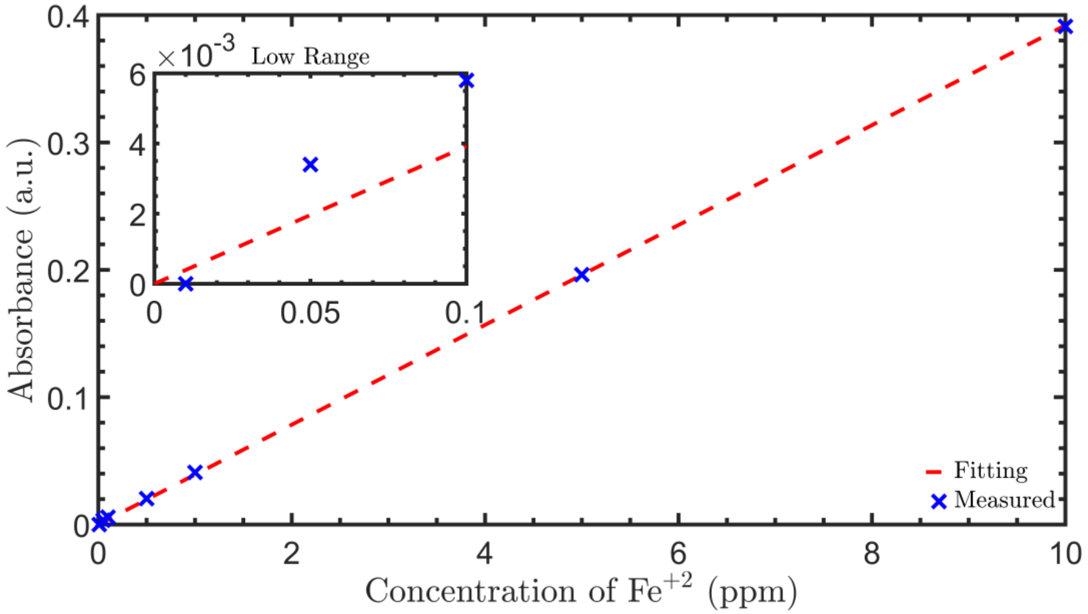

Figure S2. Calibration of ferrous Fe^{+2} for analysis using spectrophotometer.

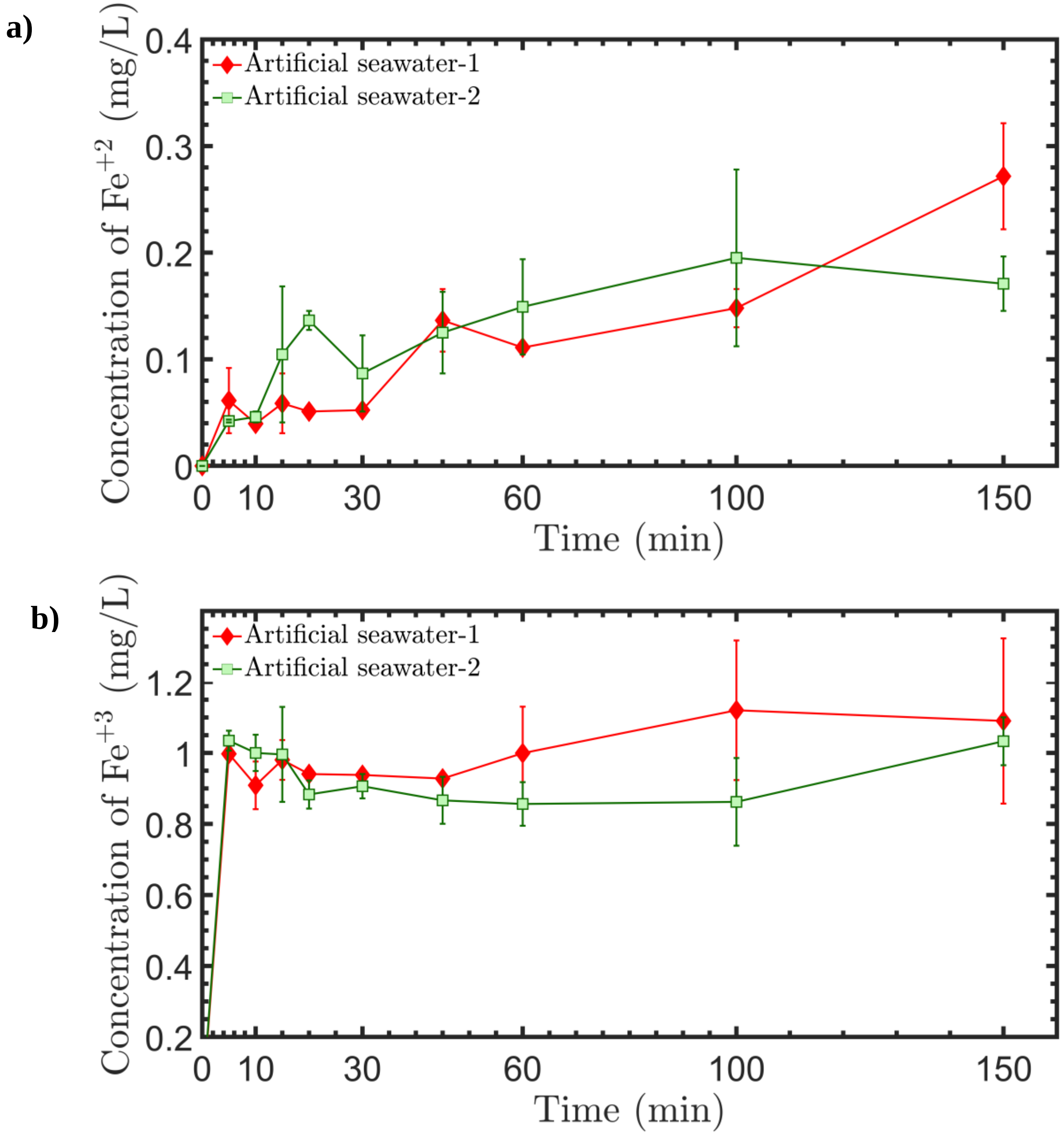

Figure S3. Concentration of a) Fe^{+2} and b) Fe^{+3} in various artificial seawater samples.

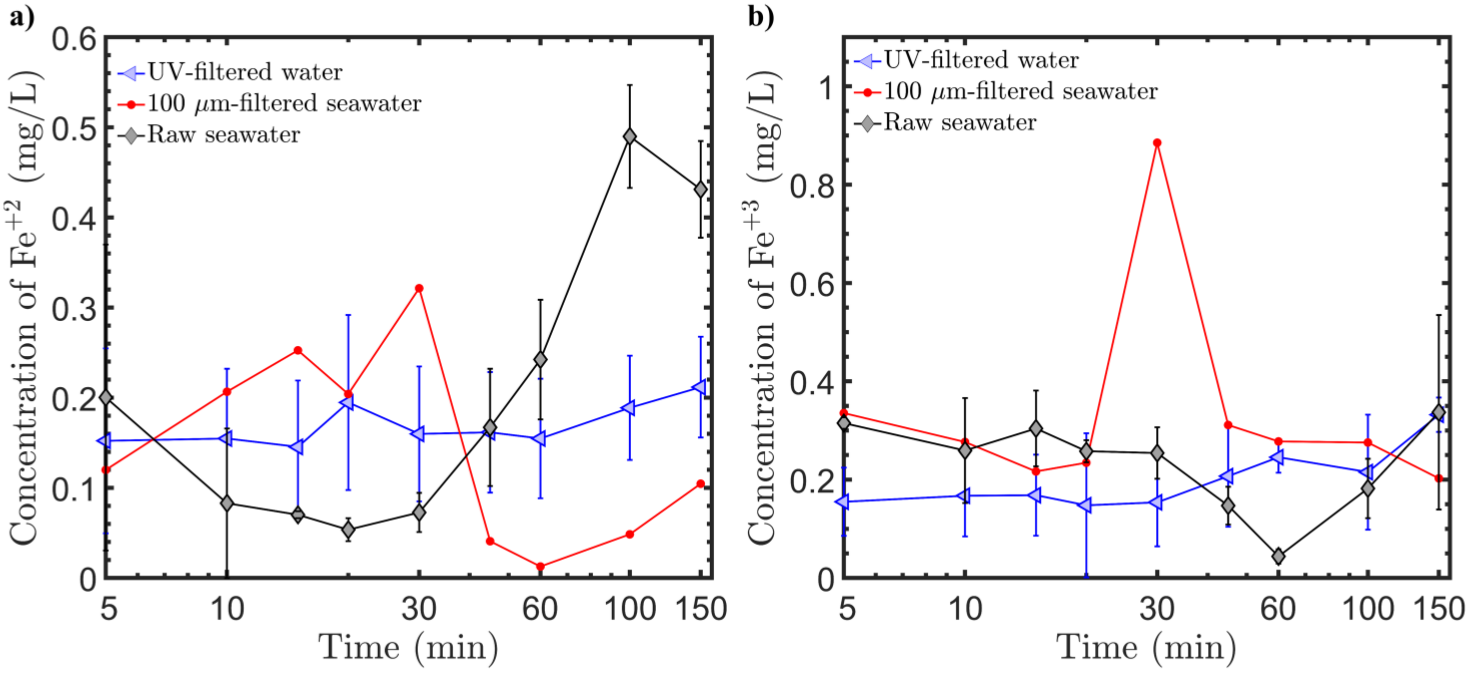

Figure S4. Concentration of iron ions in different natural seawater samples.

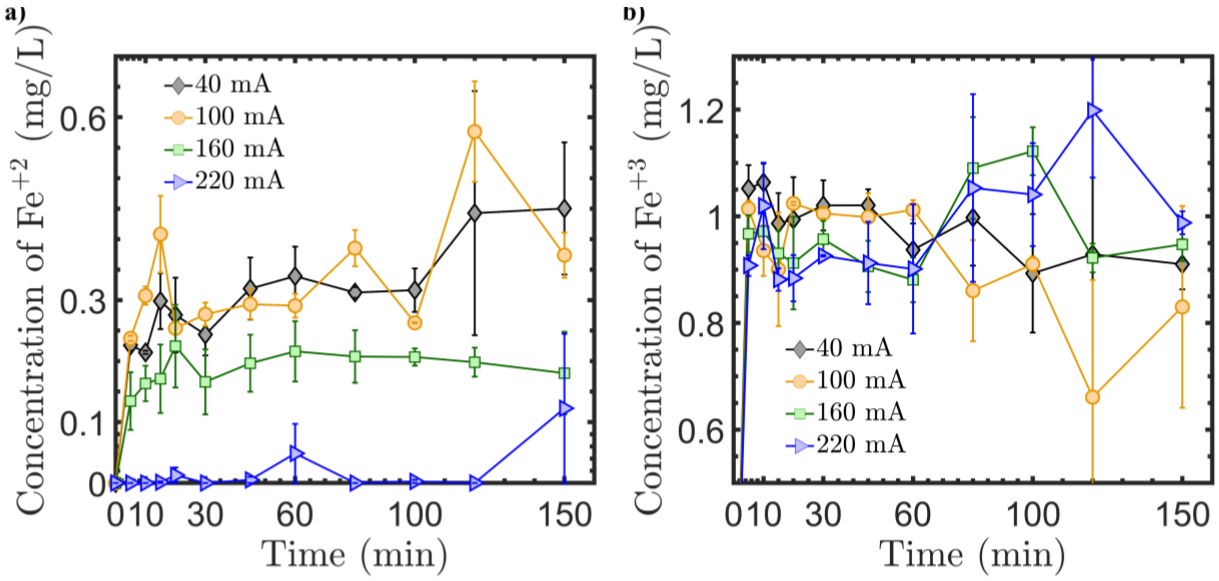

Figure S5. Concentration of iron ions for different applied currents using carbon cathode in a 1 L baker

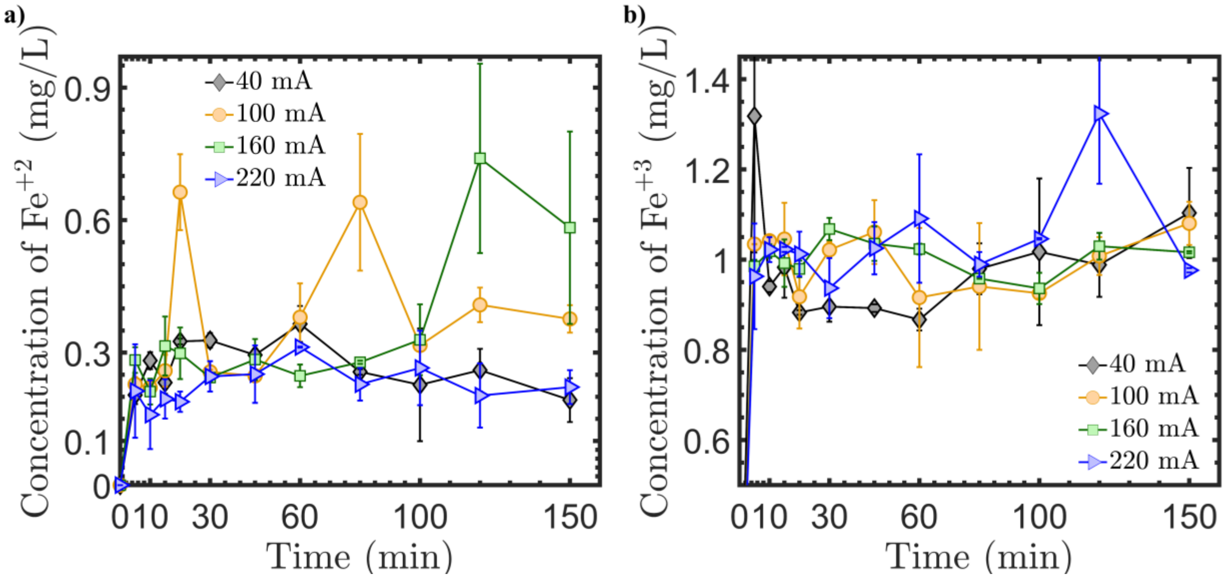

Figure S6. Concentration of iron ions for different applied currents using Ti/MMO cathode in a 1 L baker
