# Supplementary figures and images for: Electrochemical ocean iron fertilization and alkalinity enhancement approach toward CO2 sequestration
Source: NPJ Ocean Sustain. Author manuscript; Available in PMC 2024 Dec 13. (PMC11643492; doi:10.1038/s44183-024-00064-8)

## Slide 1
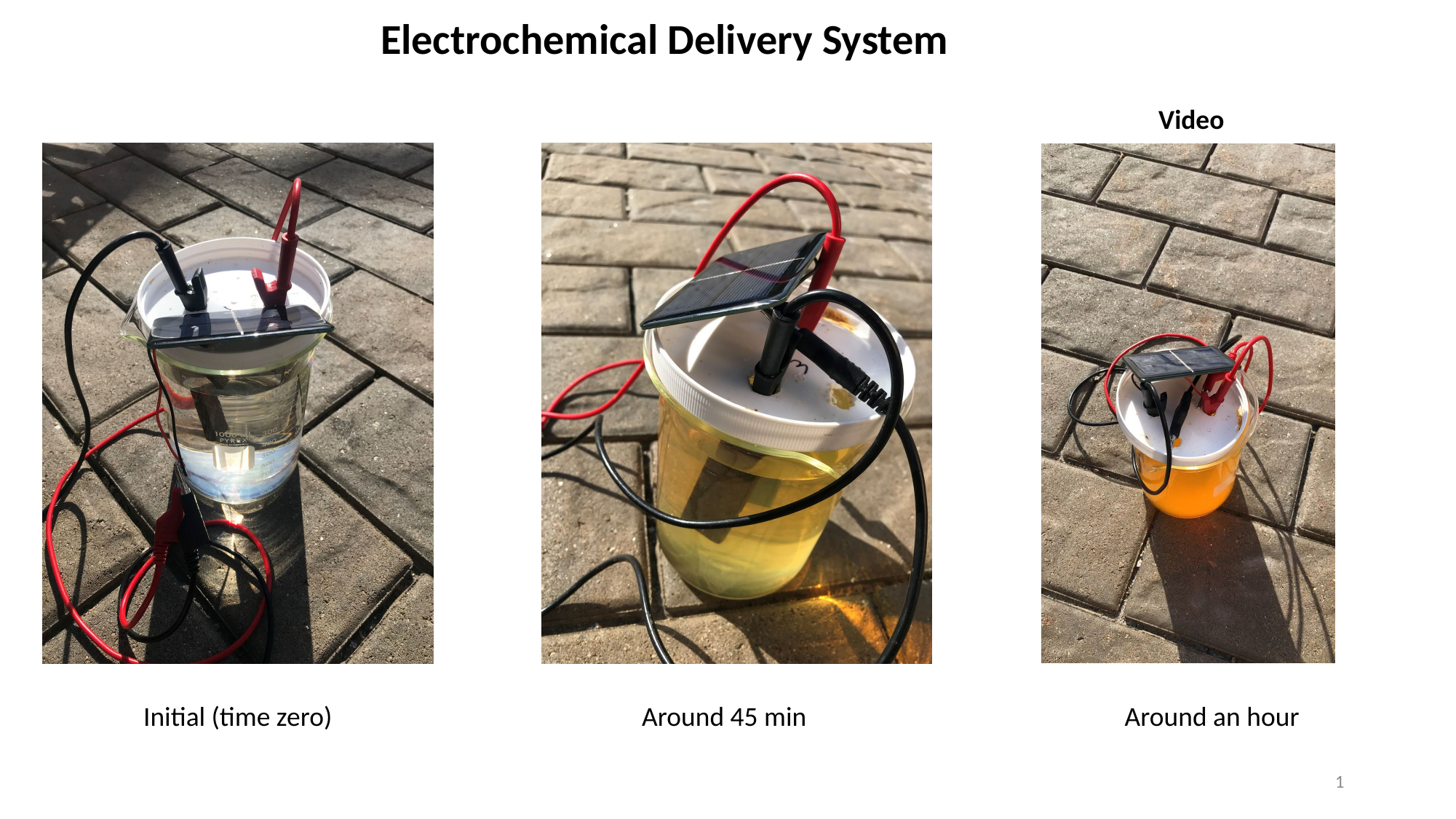

Electrochemical Delivery System
Video
Initial (time zero)
Around 45 min
Around an hour
1

Supplement: SI Video PPT [file NIHMS1995114-supplement-SI_Video_PPT.pptx]
